# Influence of Tools and Cutting Strategy on Milling Conditions and Quality of Horizontal Thin-Wall Structures of Titanium Alloy Ti_6_Al_4_V

**DOI:** 10.3390/s23249905

**Published:** 2023-12-18

**Authors:** Szymon Kurpiel, Bartosz Cudok, Krzysztof Zagórski, Jacek Cieślik, Krzysztof Skrzypkowski, Witold Brostow

**Affiliations:** 1 Faculty of Mechanical Engineering and Robotics, AGH University of Science and Technology, Mickiewicza 30 Av., 30-059 Krakow, Polandzagkrzys@agh.edu.pl (K.Z.);; 2 Faculty of Civil Engineering and Resource Management, AGH University of Science and Technology, Mickiewicza 30 Av., 30-059 Krakow, Poland; 3 Laboratory of Advanced Polymers & Optimized Materials (LAPOM), Department of Materials Science and Engineering, University of North Texas, 3940 North Elm Street, Denton, TX 76207, USA; brostow@unt.edu; 4 Department of Physics, University of North Texas, 3940 North Elm Street, Denton, TX 76207, USA

**Keywords:** horizontal thin-walled element, milling, titanium alloy, Ti_6_Al_4_V, aerospace material, acceleration vibration, short-time Fourier transform, waviness, roughness

## Abstract

Titanium and nickel alloys are used in the creation of components exposed to harsh and variable operating conditions. Such components include thin-walled structures with a variety of shapes created using milling. The driving factors behind the use of thin-walled components include the desire to reduce the weight of the structures and reduce the costs, which can sometimes be achieved by reducing the machining time. This situation necessitates, among other things, the use of new machining methods and/or better machining parameters. The available tools, geometrically designed for different strategies, allow working with similar and improved cutting parameters (increased cutting speeds or higher feed rates) without jeopardizing the necessary quality of finished products. This approach causes undesirable phenomena, such as the appearance of vibrations during machining, which adversely affect the surface quality including the surface roughness. A search is underway for cutting parameters that will minimize the vibration while meeting the quality requirements. Therefore, researching and evaluating the impact of cutting conditions are justified and common in scientific studies. In our work, we have focused on the quality characteristics of horizontal thin-walled structures from Ti_6_Al_4_V titanium alloys obtained in the milling process. Our experiments were conducted under controlled cutting conditions at a constant value of the material removal rate (2.03 cm^3^⁄min), while an increased value of the cut layer was used and tested for use in finishing machining. We used three different cutting tools, namely, one for general purpose machining, one for high-performance machining, and one for high-speed machining. Two strategies were adopted: adaptive face milling and adaptive cylindrical milling. The output quantities included the results of acceleration vibration amplitudes, and selected surface topography parameters of waviness (W_a_ and W_z_) and roughness (R_a_ and R_z_). The lowest values of the pertinent quantities were found for a sample machined with a high-performance tool using adaptive face milling. Surfaces typical of chatter vibrations were seen for all samples.

## 1. Introduction

The increasing demand for transportation—including that in the aerospace industry—is forcing manufacturers to increase the number of machines they have and to use designs with a higher reliability while reducing costs. Consequently, it is necessary to improve and seek new solutions in the design and manufacture of aerospace structures [1]. One of the paths to reduce manufacturing costs is the reduction of the weight of the structures while ensuring sufficient stiffness during the application of loads [2,3,4,5]. Accordingly, there is a notable interest in the aerospace industry in the use of thin-walled parts. In aerospace structures, thin-walled elements are found in the fuselage, landing gear, and bodies, among others [6]. One of the more frequently used methods for machining parts with complex shapes, including thin-walled parts, is milling; hence, there are continuous improvements not only of machining machines but also of cutting strategies and cutting tools [7]. As for the cutting tools, manufacturers design their geometry accordingly and also use new materials, in the bulk as well as for the coatings. The desired effects include a reduction in the machining time while retaining a sufficient quality of the finished products. In addition to appropriate tool selection, to obtain the required surface while reducing the time various shaping strategies are also being used during the execution of individual operations [8]. We note the emergence of new machining strategies such as adaptive milling, which is not yet widely used in the creation of thin-walled parts. This milling strategy allows for a significant increase in the machining efficiency through a spiral entry inside the pocket and a radial entry from the outside, as well as a rapid return over the workpiece to the beginning position [9]. Adaptive milling generates less heat and vibration in the cutting zone, which benefits the tool’s service life [10].

The use of newer tools and cutting strategies that enable more efficient operation can have a beneficial effect on the reduction in machining time; this translates into economic advantages: a cheaper process cost, and ultimately a lower cost of the finished product. A reduced machining time can be achieved by increasing the amount of material removed during successive passes. We need to keep in mind that this approach can have an adverse effect on machining conditions, including an increase in the vibrations caused by moving parts [11], large changes to the surface roughness [3,12], and significant changes to the tool’s service life [13]. Cutting parameters, machining strategies, and the cutting tools used are just some of the factors that affect the generation of vibrations [14].

The control of vibrations during machining is carried out by measuring the vibrations of the system, recording them in the form of a signal, and then analyzing the results using mathematical tools. A key tool in signal analysis, as well as in the control of the machining process, is the Fourier transform [15,16,17]. One of the most widely used tools for frequency analysis tools is the Fast Fourier Transform, which is a mathematical method of signal processing in which a signal is transformed from the time domain to the frequency domain [18,19]. The main problem with using the fast Fourier transform (FFT) is the inability to represent signal changes over time [20]. One alternative is the short-time Fourier transform (STFT), which allows the representation of signal changes over time. The STFT principle is based on the discrete Fourier transform (DFT) and represents the frequency and phase components of a time-dependent signal segment [21].

Thin-walled structures are even more susceptible to increased vibrations and process instability, and ultimately to surface failure due to the reduced stiffness of these components [22,23,24,25]. To evaluate the properties of the finished product, such as the surface topography, it seems useful to better understand the phenomena occurring during the machining process. We need information about the causes of inconsistencies occurring during the manufacture of the components so as to be able to improve the process. Thus, we need to measure the vibrations during machining to be able to determine whether the process is stable, and/or to note anomalies during machining that can result in surface defects [3,12,26]. Surface quality, as represented by surface roughness [5], is important here. The roughness has a significant impact on the quality of the finished products; it is related to wear resistance, fatigue strength, and assembly relations between components [27,28].

Several publications report on machining vibration analysis and surface topography control. In particular, the use of spectrogram plots to study conditions during machining, obtained using the STFT, is becoming more common. Still, this tool is not as widely used as the fast Fourier transform.

Rusinek and Lajmert [29] published time-frequency spectrograms of the resultant force for parameters in the stable and unstable machining range during the milling of carbon fiber-reinforced plastic. Liu and Jin [12] discussed using spectrograms pertaining to vibration control in the grinding process. They correlated the vibration waveforms with the surface topography. One of their conclusions was that chatter vibrations cause a significant increase in the surface roughness.

The short-time Fourier transform is also used to control the turning process. Frigieri et al. [30] described the possibility of using a spectrogram to evaluate the vibration frequency of a workpiece made of reinforced steel during the turning. A relationship between the vibration results obtained and the roughness parameters was demonstrated.

Chen et al. [2] presented spectrograms of system vibrations during the milling of a Ti6Al_4_V titanium alloy sample as a comparative tool for determining the process stability and the occurrence of chatter vibrations. Moreover, Chen et al. [3] compared various approaches of cutting force analysis, such as the Fourier transform, short-time Fourier transform, and continuous wavelet transform, to detect vibrations during the milling of an aluminum alloy sample. The use of the STFT made it possible to determine anomalies that occurred during varying machining conditions. It was observed that increasing the machining parameters resulted in the appearance of more frequency bands, indicating an increase in the vibration of the system.

Yan et al. [31] discussed tool vibrations during milling and studied the effects of process parameters on the surface roughness. Their discussion provides a basis for optimizing surface roughness parameters.

In an earlier paper [28], we discussed the possibility of using the short-time Fourier transform to analyze the acceleration vibration signal during the milling of thin-walled structures. Thin-walled samples were then machined in a vertical orientation using various side milling approaches. The results showed that the STFT can control anomalies during the milling process. In the present work, we aim to evaluate the quality characteristics of the finished product, as defined using selected surface topography parameters (waviness and roughness) and their correlation with machining conditions defined using vibrations occurring during milling. As part of the ongoing research, the samples will be machined with a thin wall in a horizontal orientation under controlled machining conditions using an increased cross-section of the cut layer and two adaptive milling approaches, which were tested for use in finish machining. Vibration spectrograms in the sample length domain will be created for the components of acceleration amplitudes using the short-time Fourier transform, as well as histograms showing the distribution of selected surface topography parameters in the assumed measurement areas. In the implementation of such a project, we need to pay attention to several factors:

- The use of vibration spectrograms for the control of the machining process seems feasible [2,3,12,28,29,30,31]. The publications just cited lead us to the conclusion that the use of such a signal processing method can be the basis for the process control and detection of anomalies during material processing.
- Verifying the applicability of adaptive milling when machining thin-walled structures under predefined cutting conditions is going to be valuable in future research involving this machining strategy [9].
- A literature review seems to show insufficient attention to thin-walled structures in the horizontal orientation. Most papers deal with such structures in the vertical orientation [8,32,33,34,35,36].

## 2. Materials and Methods

We studied thin-walled samples of Ti_6_Al_4_V titanium alloy in the horizontal orientation. The input factors included tools used and a cutting strategy. We used three different cutting tools (monolithic cutters) dedicated to three different machining methods (for general purpose machining, high-performance machining, and high-speed machining) and two machining strategies: adaptive face milling in the first case, and adaptive cylindrical milling in the second case. The output factors pertained to the machining process and to the finished products. The machining process was characterized using spectrograms of vibration signals prepared using the short-time Fourier transform and related to the length of each sample. The surfaces of the thin-walled structures were characterized using surface topography parameters of waviness and roughness.

### 2.1. Machining Conditions

**Figure 1:**
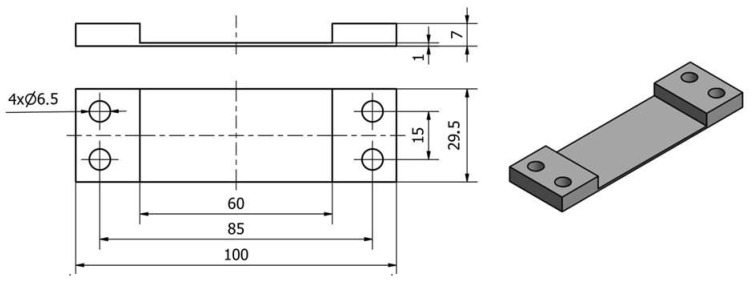
A 3D model of a thin-walled sample in the horizontal orientation.

We worked with thin-walled samples in the horizontal orientation. A horizontal wall of 1.0 mm thickness was made across the entire width of each sample (equal to 29.5 mm) and the length of 60.0 mm; see Figure 1. An 8 mm thick sheet of metal was used for the samples, which was ground (on both sides) to a dimension of 7.0 mm using a SGA3063AHD surface grinder manufactured in Poland by the company Jazon (Białystok, Poland). Then, 30 × 101 cubes were cut using a WaterJet HWE-P 1520 machine tool supplied by the German company H. G. Ridder Automatisierungs GmbH (Hamm, Germany). In the final step, the bar was milled to a dimension of 29.5 × 100 mm and mounting holes of ∅6.5 were drilled (for mounting in the adapter) on a DMC 1150 V machine (manufactured and provided by German company DMG Mori (Wernau, Germany)).

The following cutting tools (monolithic milling cutters) from Seco Tools designed for three different machining methods were used for sample preparation:

- Tool 1: JS554100E2R050.0Z4-SIRA: for general purpose (for machining all materials) [37];
- Tool 2: JS754100E2C.0Z4A-HXT: tool for high-performance machining (dedicated to machining titanium alloys and nickel superalloys) [38];
- Tool 3: JH730100D2R100.0Z7-HXT: tool for high-speed machining (dedicated to the machining of titanium alloys and nickel superalloys) [39].

**Figure 2:**
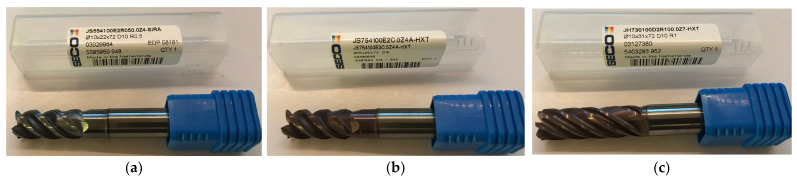
Monolithic milling cutters used to prepare samples: (**a**) Tool 1: JS554100E2R050.0Z4-SIRA; (**b**) Tool 2: JS754100E2C.0Z4A-HXT; (**c**) Tool 3: JH730100D2R100.0Z7-HXT.

Monolithic milling cutters were chosen so as to provide similar cutting parameters and to enable identical workpiece shaping operations. We used milling cutters with a single diameter of 10.0 mm. Figure 2 shows the cutters used.

**Figure 3:**
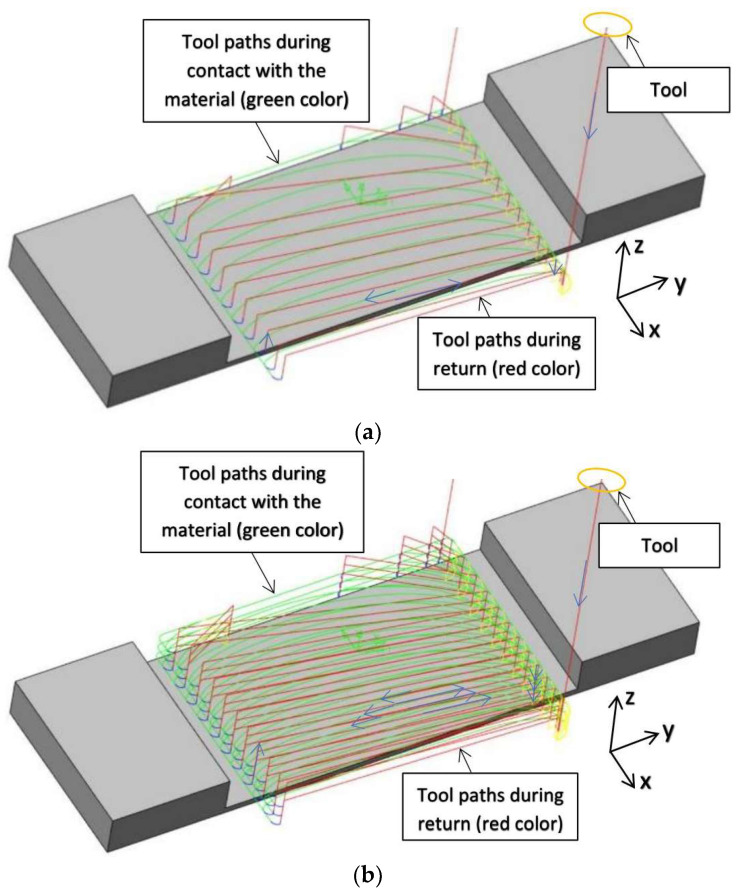
Milling paths: (**a**) adaptive face milling (large radial depth); (**b**) adaptive cylindrical milling (large depth of the cut).

The second pertinent input factor was the cutting strategy. Two milling strategies were used to make samples containing a horizontal wall: face and cylindrical. In the case of the first strategy (face adaptive milling), a relatively large value of the radial depth a_e_ equal to 4.0 mm was used to engage the face part of the cutter sufficiently, while the depth of the cut a_p_ was equal to 2.0 mm. In the case of the second strategy (adaptive cylindrical milling), a larger value of depth of cut a_p_ (equal to 6.0 mm) was used, with the aim to achieve greater involvement of the cylindrical part of the milling cutter, and the radial depth a_e_ was equal to 1.33 mm. Figure 3 shows the milling paths used during machining samples with horizontal thin walls. Blue arrows show the direction of tool movement in successive passes.

Comparison of the measured output factors of the finished products was possible due to the use of a constant material removal rate (MRR) and identical machining conditions (workpiece material, type of coolant, cutting parameters, method of workpiece clamping, experimental and measuring instrumentation). We kept a single MRR value equal to 2.03 cm^3^⁄min. The material removal rate depends on the feed rate V_f_ (mm/rev), depth of the cut a_p_ (mm), and radial depth a_e_ (mm) [5,28,40,41]. This was calculated as follows:

MRR = V_f_·a_p_·a_e_ [cm^3^/min] (1)

A typical aerospace material Ti_6_Al_4_V Grade 5 was used because of its extensive application in aerospace structures including thin-walled components [42,43,44,45]. Pertinent mechanical properties and the chemical composition of that alloy Ti_6_Al_4_V are provided in Table 1 and Table 2. In all tables in this paper, numbers with more than three digits should not be considered as containing significant digits only.

Constant parameters and cutting conditions listed in Table 3 were used to machine thin-walled samples in the horizontal orientation. The machining conditions and cutting parameters were selected from the catalog of the cutting tool manufacturer (SECO Tools) [37,38,39]. We applied parameters from the middle of the acceptable ranges.

Additional conditions occurring during the machining of the samples were the installation of monolithic milling cutters in a ∅10-precision bushing and those placed in an ER32 tool holder. Machining was carried out using coolant according to the cutting tool manufacturer’s recommendations. For milling the samples, SILUB MAX water–oil emulsion was used, which is a two-component coolant. According to the coolant manufacturer’s recommendations, a mixture of 15% oil emulsion and 85% water was used during machining [46].

**Figure 4:**
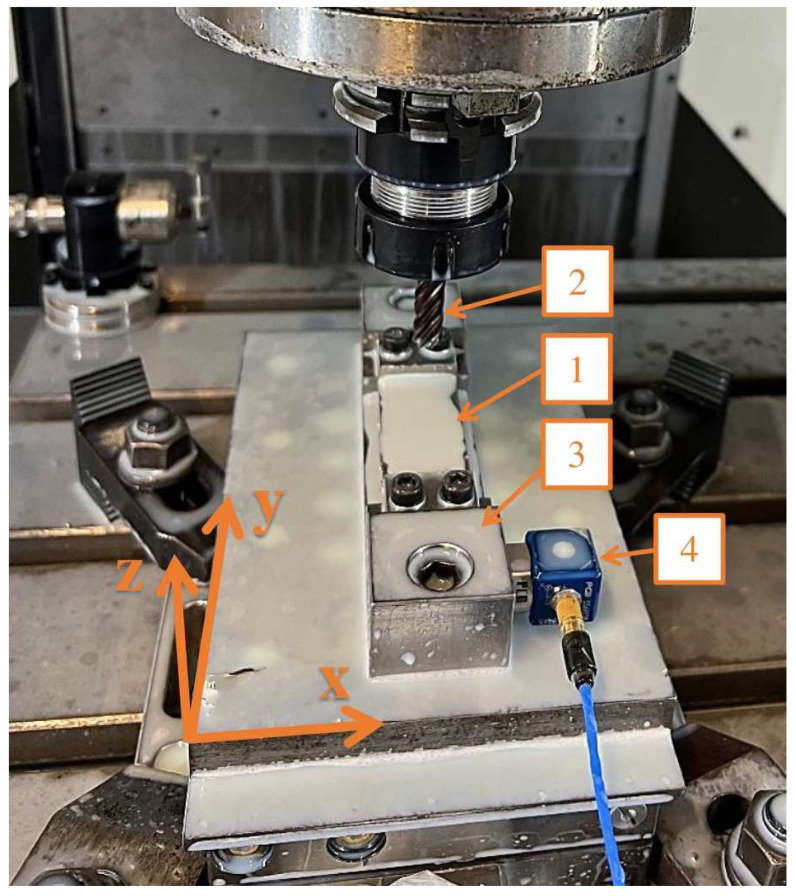
Experimental setup used during milling samples with horizontal thin walls: 1—sample, 2—tool, 3—adaptor, 4—vibration sensor.

The experiment was carried out on the milling machine Mikron VCE 600 Pro manufactured by GF Machining Solutions (Biel/Bienne, Switzerland) with control software iTNC 530 developed by Heideinhain (Traunreut, Germany). Cubes for machining samples with horizontal thin wall were mounted to the prepared adaptor, which was bolted to the threaded holes of the dynamometer using two M10 screws. The dynamometer was mounted to the machining center table using a dedicated mounting method. The experimental setup for sample processing is shown in Figure 4.

### 2.2. Measurement of Vibration Signal

The vibration signals were measured using a vibration sensor PCB-356B08 (manufactured by PCB Piezotronics Inc., Depew, NY, USA) connected to a measurement card NI USB-9162; the card transferred the signal to a pre-prepared measurement program in the LabView environment. During sample preparation, the vibration sensor was mounted on an adaptor; the method of mounting the sensor is shown in Figure 4. Based on the resulting measurement signal, spectrogram graphs were prepared using the spectrogram function with Hanning window implemented in MatLAB R2022B. The signal from the last full pass for each sample was used to generate graphs. The subsequent parameters depended on a ‘framelen’ parameter equal to 471, which is the number of samples per one full period of oscillation (derived from the rotational motion of the spindle). Four times the ‘framelen’ was assumed for the Hanning window settings, while the overlap was half the value selected for the window. Equation (2) shows the line from MatLAB used to prepare the spectrogram.

[Sw, W] = spectrogram(signal, hann(4·framelen), 2·framelen, 4·framelen, fs)(2)

Spectrogram plots are presented in the sample length domain using Equation (3) implemented in a dedicated program:

S = V_f_·t [mm] (3)

In the above Equations (2) and (3), ‘signal’ means the vibration signal measurement data, V_f_ is the feed rate (a constant value of 255 mm/min was applied), t is the machining time, and f_s_ is the sampling frequency equal to 25,000 Hz.

The frequency measurement range of the vibration sensor used was 0.5–5000 Hz. In the conducted experiment, no significant results were observed in the range of 2000–5000 Hz; hence, this range was not included in the graphs.

### 2.3. Measurement of Surface Topography

The measurement of surface topography parameters was determined using the contact profilometer Topo 01P v3D (Poland). The samples were placed on a sliding table of the MY120-AS (Poland) machine coupled to a signal recorder, and dedicated software was used to measure surface topography. 

**Figure 5:**
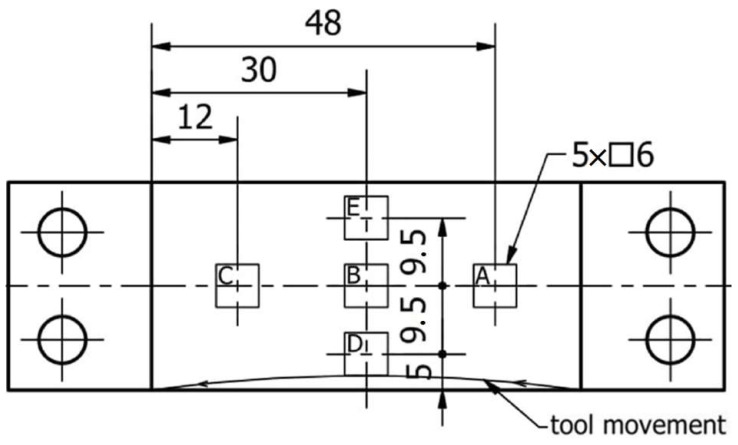
The areas of measurement of surface topography parameters.

Surface topography parameters were determined in 5 areas marked A, B, C, D, and E in Figure 5 on the machined surface. For each selected area, 13 profiles 500 μm apart with a length of 6.0 mm were measured, with a measurement step of 1 μm and a measurement speed of 0.5 mm/s. Areas A, B, and C allowed the determination of surface topography parameters perpendicular to the direction of the tool’s feed motion, while areas D, B, and E pertained to the direction parallel to the tool’s feed motion.

### 2.4. Statistical Analysis

Statistical analysis of the results obtained by measuring machining vibrations and thin-wall deviations was carried out using Statistica v13.3. The analysis includes box-plots and a table containing the basic statistics of the measured parameters. The box-plot charts include the mean and the standard deviation. The tables with basic statistics provide numerical information on the mean, median, minimum and maximum values, variance, standard deviation, and standard error.

For the samples with horizontal thin walls, we have considered the following output parameters:

- Vibration—the maximum values of the three components of the acceleration vibration amplitudes for all full passes made in the sample processing were assumed. For statistical analysis, the total amplitude of acceleration vibration A_c_ was determined using vector folding of the components on the x, y, and z axes.
- Surface topography—the values of the waviness parameters (Wa and Wz) and roughness parameters (Ra and Rz) were determined in 5 measurement areas (areas A–E), as shown in Figure 5.

## 3. Results and Discussion

The results presented in this work are part of a larger study. In an earlier paper [47], thin-wall deviations determined using contact (coordinate measuring machine) and optical methods (3D optical scanner) were reported.

### 3.1. Vibration Spectrograms

**Figure 6:**
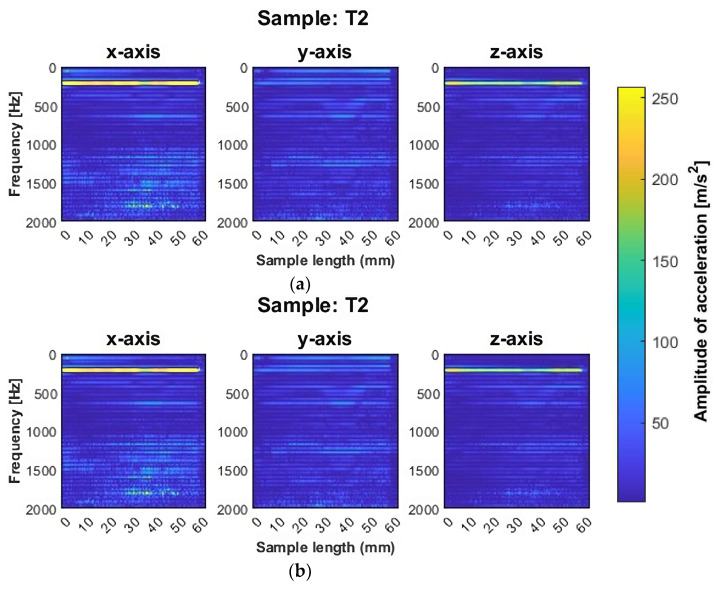

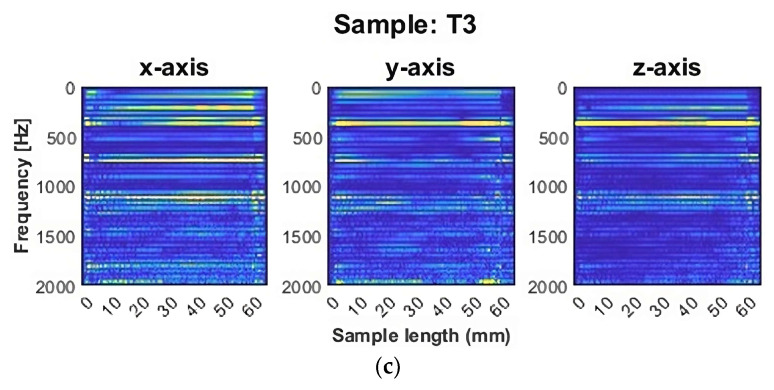
Vibration spectrogram of the acceleration signal for the sample: (**a**) T1; (**b**) T2; (**c**) T3.

**Figure 7:**
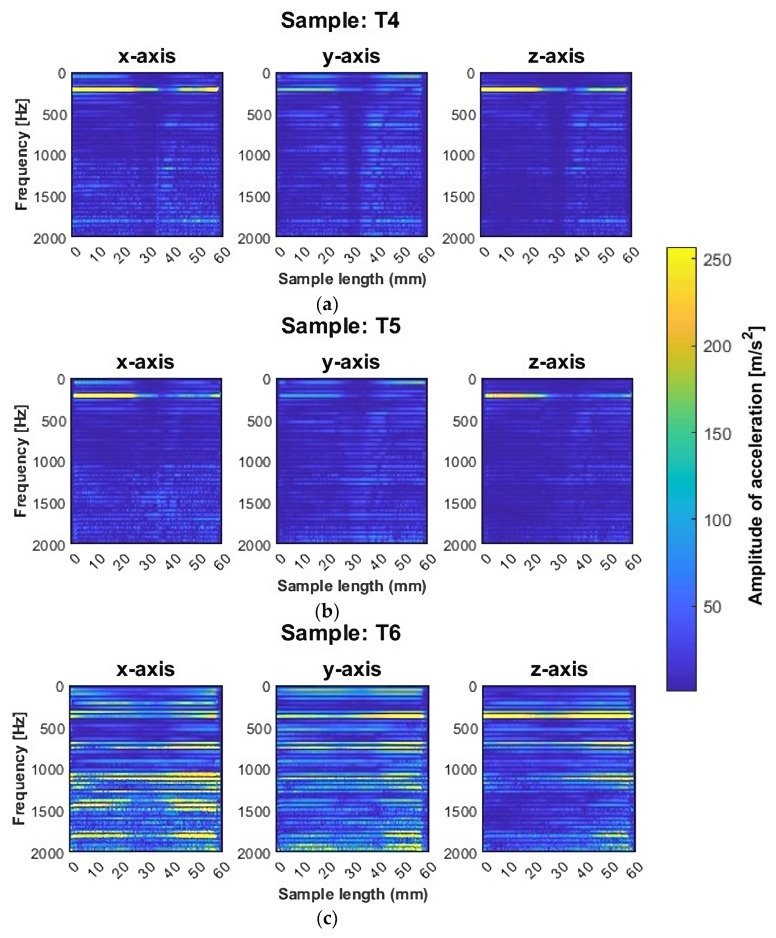
Vibration spectrogram of the acceleration signal for the sample: (**a**) T4; (**b**) T5; (**c**) T6.

We analyzed each signal from the last full pass, since such signals seemed to reflect the nature of each surface. Spectrograms for these signals were prepared as described in the previous section. Figure 6 shows the spectrograms of samples machined with adaptive face milling, while Figure 7 shows those machined with adaptive cylindrical milling.

Samples T1 and T2, milled using adaptive face milling with the tool for general purpose machining and the tool for high-performance machining, respectively, had quite similar distributions of acceleration amplitudes. This effect may be due to the similar geometries of the two tools, resulting in similar cutting conditions in terms of machining vibrations. For both samples, we found that a blurring of the frequency bands appeared after reaching half of the sample length, i.e., in the range from approximately 30 mm to approximately 50 mm, with no tendency to increase the values of the amplitudes. The blurring of the bands was particularly observed on the y-axis (the direction of the feed motion), while it also occurred on the z-axis with a lower intensity. This may indicate the occurrence of vibrations during machining under such conditions. For both samples (T1 and T2), a high amplitude frequency band was observed near 212 Hz (on the x and z axes), which is the value of the fourth harmonic of vibration. For the T3 sample, milled with the tool for high-speed machining, and also machined with adaptive face milling, the blurring of bands that occurred for the previous samples after crossing the center of the sample was not seen. However, a significant number of frequency bands appeared on the T3 sample spectrogram. Their location on the spectrogram indicated the presence of harmonic oscillations with a wide frequency range. In addition, it is noticeable that the waveforms of the graphs are not stable; there are a variety of vibration amplitudes in the bands. We infer that the processing under such conditions was not stable.

A similar distribution of plots between the corresponding components, as in the case of samples T1 and T2, was also noted for samples made with the same tools but using adaptive cylindrical milling (T4 and T5). For this strategy (adaptive cylindrical milling), a band for frequencies around 212 Hz also appeared both on the y and z axes, as well as on the x-axis (this was not the case for the T1 and T2 samples). In addition, a decrease in amplitude can be seen after reaching half of the sample length (about 30 mm), along with a blurring of the frequency bands from approximately 30 mm to approximately 50 mm, as in the case of samples T1 and T2 (made with the same tools, but with the opposite strategy).

For the T4 and T5 samples, the intensity of this phenomenon varied depending on the machining tool used. For the T4 sample, a decrease in the amplitude in the band for a frequency around 212 Hz is visible, followed by its increase and stabilization. For the T5 sample, after a decrease in amplitude, its increase to the previous value was not observed. In both cases, this may be due to a change in the direction of feed motion, and therefore a change in the involvement of the peripheral part of the tool during machining.

For the T6 sample, machined with the tool for high-speed machining using adaptive cylindrical milling, the tendency for a decrease after reaching half of the sample length was not as pronounced as for the other samples made with the same strategy (T4 and T5). The T6 sample shows some similarities in signal to the T3 sample, the latter made with the same tool but using the opposite strategy. It was also noticeable that there were frequency bands indicative of harmonic oscillations, but for the T6 sample such bands seemed to appear at higher frequency values. The distribution of such bands was also uneven along the length of the sample; hence, we conclude that the machining process was also unstable under such conditions. In summary, for the samples made with the tool designed for high-speed machining (T3 and T6), it is apparent, based on the color scale, that there were more frequencies present compared to samples machined with other tools. Apparently there were stronger vibrations in these samples when using this cutter. The titanium alloy samples made with the tool for high-speed machining (T3 and T6) showed the occurrence of distinct frequency bands characteristic of harmonic vibrations over the entire range studied.

Based on the spectrograms shown in Figure 6 and Figure 7 for titanium alloys, it is not possible to determine which sample or strategy led to the smallest anomalies. Further consideration of this issue will be carried out in the statistical analysis of the results. Based on the presented graphs, it can be reported that the use of the tool for high-speed machining (T3 and T6) yields undesirable machining conditions, manifested by the significant number of frequency bands present and their large amplitude values, also for harmonic frequencies.

Based on visual observations, the surfaces characteristic for chatter vibration were seen for titanium alloy samples (T1–T6); this is also reflected in the instabilities seen in Figure 6 and Figure 7.

### 3.2. Surface Topography

The parameters pertaining to the surface topography of samples containing thin horizontal walls were determined on the machined surfaces in selected measuring areas, marked according to Figure 5; the measurements were carried out using the methodology described in Section 2.3.

**Figure 8:**
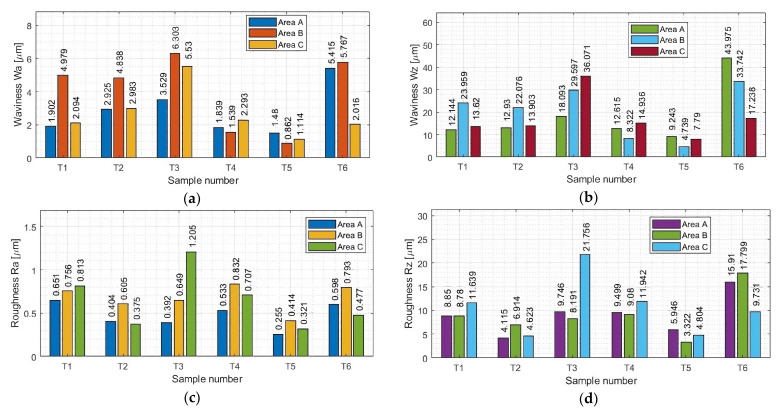

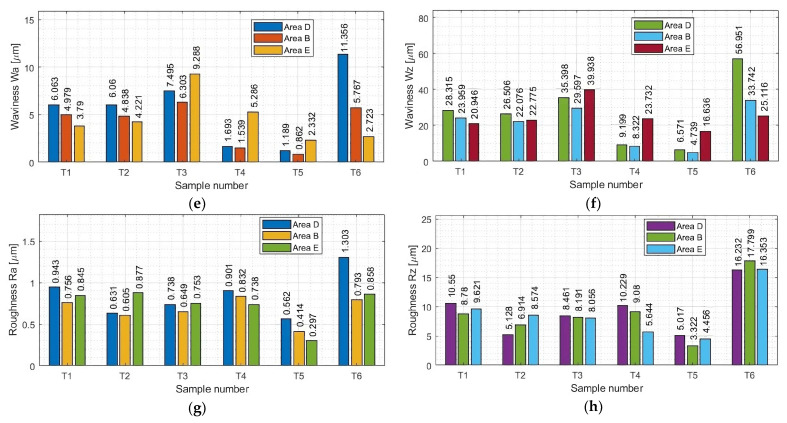
Values of (**a**) Wa in areas A, B, and C; (**b**) Wz in areas A, B, and C; (**c**) Ra in areas A, B, and C; (**d**) Rz in areas A, B, and C; (**e**) Wa in areas D, B, and E; (**f**) Wz in areas D, B, and E; (**g**) Ra in areas D, B, and E; (**h**) Rz in areas D, B, and E.

First, measurements were made for the areas A, B, and C (parallel to the direction of feed motion), followed by measurements for the areas D, B, and E (perpendicular to the direction of feed motion). In our work, area B is common and located in the center of the sample. A summary of the results pertaining to the surface topography is provided in Appendix A in Table A1 and Table A2. Based on the data contained in these tables, we have created graphical representations (in the form of histogram plots) of the arithmetic mean waviness W_a_, the maximum height of the waviness W_z_, the arithmetic mean deviation R_a_, and the maximum height R_z_. The results pertaining to the waviness and the roughness in areas A, B, and C are shown in Figure 8a–d, while those for areas D, B, and E are shown in Figure 8e–h.

From the waviness graphs of Wa and Wz in Figure 8a,b,e,f, the similar nature of the distribution of the plots between the two parameters can be noted; apparently these parameters are dependent on each other. Based on the graphs presented, it is not possible to clearly determine which strategy led to lower values of the waviness parameters. Although the waviness values Wa and Wz seem to be smaller for the samples milled using adaptive cylindrical milling (T4–T6) than for those made with adaptive face milling (T1–T3), a lack of repeatability and stability is seen. For the adaptive cylindrical milling samples (T4–T6), we see an increase in the values of the waviness parameters, mainly in the E area, that is, within the zone of the aforementioned run-out noted above. An additional issue affecting the waviness in this area is the fact that the bulge occurred during the last full pass, where the sample lost its stiffness because of the re-removal of material in the center of the sample. This resulted in an increase in the value of the waviness Wz, manifested by the deflection of the sample, which also caused an increase in the value of the waviness Wa. We need to keep in mind that the values of these parameters in each area should be smaller than the assumed critical value. Even though the other areas have much smaller waviness values, the occurrence of anomalies in individual areas can have an adverse effect on the functionality and durability of the product. Somewhat unusual values in area E may be the result of a poorly chosen strategy, with a significant impact on the results of the adaptive cylindrical milling.

In the case of adaptive face milling (T1–T3), it was observed that the results in the direction parallel to the tool feed direction (areas A, B, and C) were characterized by more stable values in each area compared to the values obtained in the perpendicular direction (areas D, B, and E), and a significant deviation in the values obtained in area B from those in areas D and E is evident. This seems to be a consequence of the largest deflection in the thin wall occurring in its central part between the mounting points.

Considering the presented Wa and Wz waviness plots for all the tested samples, the smallest values were seen for the T5 sample milled with the tool for high-performance machining using adaptive cylindrical milling. For the sample machined using the opposite strategy and the same tool (T2), the waviness values were up to five times greater, and similar to those obtained with the tool for general purpose machining (T1). Samples milled with the tool for high-speed machining (T3 and T6) presented the largest values of Wa and Wz waviness parameters in their strategies (i.e., comparing samples T1, T2, and T3 with each other and comparing samples T4, T5, and T6 with each other) compared to the other samples. The results for samples T3 and T6 were unstable and irregular, with no apparent dependence in their distribution.

We now consider the Ra and Rz roughness results shown in Figure 8c,d,g,h. The lowest values of the waviness parameters were seen for sample T5, which was made with the tool for high-performance machining using adaptive cylindrical milling. The surface of the sample obtained with the same tool (for high-performance machining), but with adaptive face milling (T2) was also characterized by relatively small values of roughness parameters in the group of samples made with the same strategy (i.e., samples T1–T3).

The values of roughness parameters were similar for samples machined with the tool for general purpose machining (T1 and T4). For this tool, it is concluded that the strategy used had no significant effect on the roughness parameters. The roughness values for samples machined with the tool for general purpose machining (T1 and T4) were about half those for the Ra parameter and about twice as large as those for the Rz parameter compared to their counterparts machined with the tool for high-performance machining (T2 and T5).

The highest maximum roughness values were seen when we used the tool for high-speed machining (T3 and T6). For the T3 sample, the increase in parameters was observed in area C, which was in the damaged post-treatment surface of the sample. The surface in this area showed signs of overheating of the material, possibly as a result of the blades being sealed by chips and not properly removed. In the case of the T6 sample, the peak in the histogram was observed in area D, which is in the initial part of the sample when the tool started the machining process.

Looking at the totality of our results, it is not possible to say clearly which cutting strategy—adaptive face milling or adaptive cylindrical milling—leads to lower roughness values. Considering both the waviness and roughness results obtained, we see that among the titanium alloy samples the lowest values pertain to sample T5, which was milled with the tool for high-performance machining using adaptive cylindrical milling. The highest values of these parameters were seen for sample T6, which was milled with the tool for high-speed machining using the same strategy.

### 3.3. Statistical Analysis of Results

**Figure 9:**
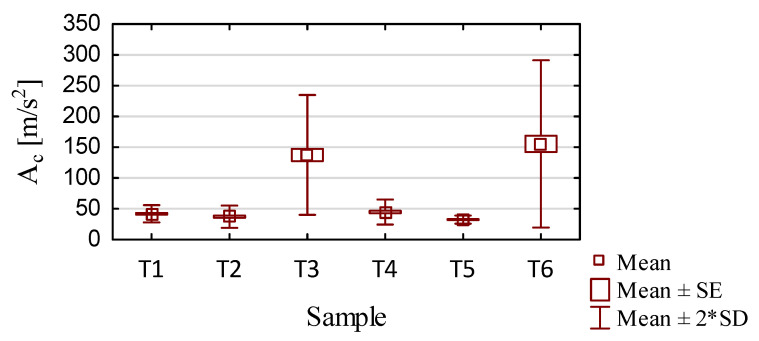
Results of acceleration vibration statistics for different samples.

**Figure 10:**
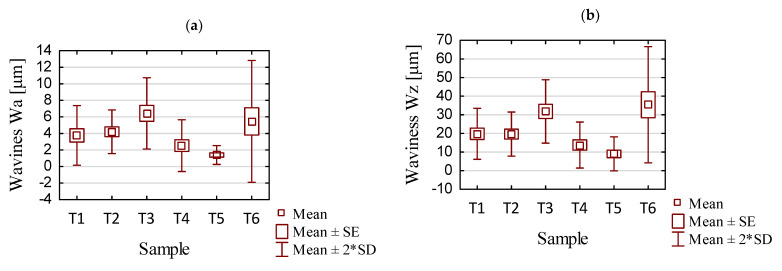

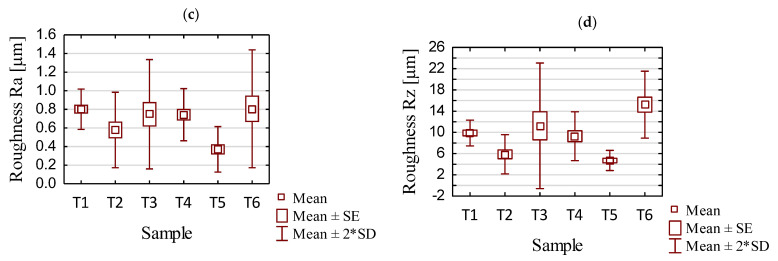
Statistical results for the processing of different samples: (**a**) waviness Wa; (**b**) waviness Wz; (**c**) roughness Ra; (**d**) roughness Rz.

For each sample, the graphs that were generated included the mean value and the mean value, standard error, and standard deviation of the measured parameters, as shown in Figure 9 and Figure 10. Table 4 and Table 5 contain the basic statistics of the measured parameters of each sample analyzed.

Based on the data presented in Figure 9 and Table 4, the following conclusions can be formulated:

- Samples milled with the tool for general purpose machining (T1 and T4) and the tool for high-performance machining (T2 and T5) had similar average values and a similar scatter of total acceleration vibration amplitude results, likely as a consequence of the similar geometries of these tools and the identical machining conditions used for thin-walled structures. The similarity in the waveforms for these samples is also seen in the vibration spectrograms shown in Figure 6 and Figure 7.
- The most stable results were obtained for samples machined with the tool for high-performance machining with adaptive cylindrical milling, i.e., for sample T5.
- Samples machined with the tool for high-speed machining (T3 and T6) also had similar average values of the total amplitude of acceleration vibration, but they were about 10% greater when using adaptive cylindrical milling (T6) compared to adaptive face milling (T3). The use of adaptive cylindrical milling with the tool for high-speed machining (T6) showed a larger scatter of values compared to the results obtained for adaptive face milling with the same tool (T3). This indicates that the process was more unstable for the T6 sample, which is also seen in the vibration spectrogram in Figure 7.
- The values of the total amplitude of acceleration vibrations for the titanium alloy samples obtained with the tools for high-speed machining (T3 and T6) were up to four times greater than those obtained when using the other tools (samples T1, T2, T4, and T5).
- It is difficult to say unequivocally which machining strategy—adaptive face milling or adaptive cylindrical milling—has a more favorable effect on minimizing vibrations. We obtained similar average values of total vibration amplitudes for samples made with the same tools.

Based on the data in Figure 10 and Table 5 containing the results of statistical analysis for selected surface topography parameters (waviness W_a_ and W_z_; and roughness R_a_ and R_z_) for samples T1–T6, we can draw the following conclusions:

- For samples milled with the tool for general purpose machining and the tool for high-performance machining, lower average values of waviness (W_a_ and W_z_) and roughness (R_a_, R_z_) were obtained when using adaptive cylindrical milling (T4 and T5) rather than adaptive face milling (T1 and T2). This conclusion does not apply to the use of the tool for high-speed machining (T3 and T6).
- As for the tool selection, the most favorable (i.e., the lowest) surface topography parameters were obtained when using the tools for high-performance machining (T2 and T5). We also obtained low values for the variance and the standard deviation; clearly, the spread of values was not large. Comparing now the T2 and T5 samples, significantly lower values were seen for sample T5 (adaptive cylindrical milling). Taking into account all samples, this sample also had the smallest scatter of the results.
- We consider now the average waviness values W_a_ and W_z_. For samples made with the general purpose machining tool (T1 and T4), the results were very similar to those obtained with the high-performance machining tool (T2 and T5). At the same time, the average roughness values R_a_ and R_z_ obtained using the general purpose machining tool (T1 and T4) were up to two times greater than those obtained when milling with the tool for high-performance machining (T2 and T5).
- Samples milled with the tool for high-speed machining (T3 and T6) exhibited significantly greater average values of measured surface topography parameters. These values were up to three times greater than those obtained for samples made with the general purpose machining tool (T1 and T4), and up to four times greater than those for samples milled with the high-performance machining tool (T2 and T5).
- Large values for the variance and the standard deviation were observed for the samples made with the tool for high-speed machining (T3 and T6), suggesting a large amount of variability in the data. The instability of the surface topography parameters has its origin in unstable cutting conditions (i.e., vibrations), which may depend on the geometry of the tool used. We infer that such cutting conditions are not recommended for machining thin-walled structures.

## 4. Summary and Concluding Remarks

We created a series of samples from Ti_6_Al_4_V titanium alloy such that each contained a thin wall in the horizontal orientation. The assumed input factors were three cutting tools and two cutting strategies. The machining of the samples was carried out using a large cross-section of the cut layer and controlled cutting conditions. The results obtained were comparable by using a constant material removal rate (MRR).

The conclusions from our work are as follows:

- During the use of adaptive cylindrical milling, the samples were seen to shine in the last full pass, which was apparently related to the surface topography.
- The process of machining the samples with the tool for general purpose machining (T1 and T4) and the tool for high-performance machining (T2 and T5) was characterized by similar values of the total amplitude of vibration, which was influenced by the similar geometry of these tools. For the mentioned samples, the process was stable and the obtained values presented a slight scatter. However, slightly lower values of total vibration amplitude were seen when using the high-performance machining tools (T2 and T5) compared to the tool for general purpose machining (T1 and T4).
- Surfaces characteristic of chatter vibrations were seen for all machined samples.
- The lowest values of the total amplitude of vibration acceleration and selected surface topography parameters (waviness Wa and Wz; and roughness Ra and Rz) were seen for sample T5, which was machined with the tool for high-performance machining using adaptive cylindrical milling. Slightly higher values were recorded for samples prepared with the same tool, but under the opposite strategy of adaptive face milling (T2).
- Samples milled with the tool high-speed machining (T3 and T6) were characterized by significantly higher values of the pertinent parameters (total amplitude of vibration acceleration, waviness, and roughness) when compared to samples prepared with the other tools (T1, T2, T4, and T5). The results for samples milled with the tool for high-speed machining showed a significant scatter of values, which is clearly an undesirable phenomenon.
- Lower values of pertinent parameters were obtained during machining with adaptive cylindrical milling than for adaptive face milling. It is not possible to say unequivocally which strategy provides more favorable results in terms of vibration minimization; there were similar values obtained using different strategies for samples made with the same tools.

We succeeded in creating thin-walled samples in the horizontal orientation. We note, however, that chatter vibrations appeared, negatively affecting the surface quality. Therefore, it would be advisable to focus on improving the processing conditions that would eliminate the occurrence of chatter vibrations.

As noted in the beginning of this paper, thin-walled structures have a variety of applications, including in aerospace materials and materials subjected to harsh and variable environments. Teaching Materials Science and Engineering (MSE) includes biomaterials [48], amorphous and glassy structures [49,50] and water purification [51,52]. It is possible that thin-walled structures also deserve some space in MSE instruction.

## Figures and Tables

**Table 1:** Mechanical properties of Ti_6_Al_4_V Grade 5 [28,41,42]).

Mechanical Properties	Value
Tensile strength R_m_ [MPa]	min. 760
Yield strength [MPa]	min. 380
Elongation at break [%]	min. 35
Density [g/cm^3^]	8.44
Melting point [°C]	1648

**Table 2:** The chemical composition of titanium alloy Ti_6_Al_4_V Grade 5 (based on [28,41,42]).

Element	Ti	Al	V	Fe	O	C
Percentage [%]	other	5.5–6.75	3.5–4.5	max. 0.4	max. 0.2	max 0.08

**Table 3:** Cutting parameters during milling thin-walled samples in horizontal orientation.

Material	Sample Number	Tool	Machining Strategy	Cutting Speed V_c_ [m/min]	Feed Rate V_f_ [mm/min]	Depth of Cut a_p_ [mm]	Radial Depth a_e_ [mm]
Titanium alloy Ti_6_Al_4_V-grade 5	T1	Tool 1	adaptive face milling	40	255	2 (3 passes)	4
	T2	Tool 2		40	255	2 (3 passes)	4
	T3	Tool 3		40	255	2 (3 passes)	4
	T4	Tool 1	adaptive cylindrical milling	40	255	6 (1 pass)	1.33
	T5	Tool 2		40	255	6 (1 pass)	1.33
	T6	Tool 3		40	255	6 (1 pass)	1.33

**Table 4:** Summary of statistical analysis of acceleration vibration results.

Sample	Mean	Median	Min.	Max.	Variance	Std. Dev.	Std. Error
T1	41.88	41.14	32.50	58.26	49.01	7	1.43
T2	37.19	35.4	26.63	62.62	80.79	8.99	1.83
T3	137.4	124.3	79.94	270.7	2369	48.68	9.94
T4	44.72	41.90	38.22	78.43	103	10.15	1.99
T5	32.42	32.21	27.89	42.87	11.07	3.33	0.65
T6	155.4	127.8	96.22	339.3	4607	67.88	13.31

**Table 5:** Summary of statistical analysis of selected surface topography results.

Parameter	Sample	Mean	Median	Min.	Max.	Variance	Std. Dev.	Std. Error
Wa	T1	3.77	3.79	1.9	6.06	3.25	1.8	0.81
	T2	4.21	4.22	2.93	6.06	1.74	1.32	0.59
	T3	6.43	6.3	3.53	9.29	4.64	2.15	0.96
	T4	2.53	1.84	1.54	5.29	2.45	1.57	0.7
	T5	1.4	1.19	0.86	2.33	0.32	0.57	0.25
	T6	5.46	5.42	2.02	11.36	13.55	3.68	1.65
Wz	T1	19.8	20.95	12.14	28.32	46.98	6.85	3.07
	T2	19.64	22.08	12.93	26.51	35.21	5.93	2.65
	T3	31.82	35.4	18.09	39.94	72.54	8.52	3.81
	T4	13.76	12.62	8.32	23.73	38.13	6.17	2.76
	T5	9	7.79	4.74	16.64	20.97	4.58	2.05
	T6	35.4	33.74	17.24	56.95	244.1	15.62	6.99
Ra	T1	0.8	0.81	0.65	0.94	0.01	0.11	0.05
	T2	0.58	0.61	0.38	0.88	0.04	0.2	0.09
	T3	0.75	0.74	0.39	1.21	0.09	0.29	0.13
	T4	0.74	0.74	0.53	0.9	0.02	0.14	0.06
	T5	0.37	0.32	0.26	0.56	0.01	0.12	0.05
	T6	0.81	0.79	0.48	1.3	0.1	0.32	0.14
Rz	T1	9.89	9.62	8.78	11.64	1.47	1.21	0.54
	T2	5.87	5.13	4.12	8.57	3.4	1.84	0.82
	T3	11.24	8.46	8.06	21.76	34.99	5.92	2.65
	T4	9.28	9.5	5.64	11.94	5.32	2.31	1.03
	T5	4.71	4.8	3.32	5.95	0.91	0.95	0.43
	T6	15.22	16.32	9.73	17.8	9.94	3.15	1.41

## Data Availability

Data are contained within the article.

## Appendix A

The values of waviness and roughness of horizontal thin-walled samples were measured using a contact profilometer for samples T1–T6 (Table A1 and Table A2). The following terms are used in the following tables: arithmetic mean waviness, Wa; maximum height of the waviness, Wz; skewness of the waviness profile, Wsk; maximum height of peaks of waviness profile, Wp; maximum depth of valleys of waviness profile, Wv; square mean of the waviness deviations, Wq; kurtosis of the waviness, Wku; arithmetic mean deviation, Ra; maximum height, Rz; skewness of the roughness profile, Rsk; maximum height of peaks of roughness profile, Rp; maximum depth of valleys of roughness profile, Rv; root mean square roughness, Rq; kurtosis of the roughness, Rku.

**Table A1:** Parameters of waviness for input site obtained using a contact profilometer.

Sample Number	Measuring Area	Wa [µm]	Wz [µm]	Wsk	Wp [µm]	Wv [µm]	Wq [µm]	Wku
T1	A	1.9	12.14	1.61	6.61	5.54	2.4	2.95
	B	4.98	23.96	1.4	13.26	10.7	5.9	2.19
	C	2.09	13.62	1.74	8.96	4.66	2.69	3.63
	D	6.06	28.32	1.42	17.21	11.11	7.08	2.34
	E	3.79	20.95	1.55	12.42	8.53	4.68	2.77
T2	A	2.93	12.93	1.29	7.03	5.9	3.31	1.83
	B	4.84	22.08	1.34	10.52	11.56	5.67	1.93
	C	2.98	13.9	1.41	8.11	5.79	3.58	2.21
	D	6.06	26.51	1.32	13.32	13.19	6.94	1.89
	E	4.22	22.78	1.43	10.29	12.49	5.09	2.24
T3	A	3.53	18.09	1.4	9.63	8.47	4.17	2.18
	B	6.3	29.6	1.34	15.22	14.38	7.35	1.94
	C	5.53	36.07	1.52	22.03	14.04	6.67	2.76
	D	7.5	35.4	1.35	19.74	15.66	8.7	2.02
	E	9.29	39.94	1.31	17.86	22.08	10.8	1.85
T4	A	1.84	12.62	1.54	5.05	7.57	2.27	2.72
	B	1.54	8.32	1.45	3.82	4.5	1.89	2.28
	C	2.29	14.94	1.48	6.83	8.11	2.76	2.51
	D	1.69	9.2	1.5	4.25	4.95	2.11	2.48
	E	5.29	23.73	1.3	12.12	11.61	6.04	1.82
T5	A	1.48	9.24	1.46	4.12	5.12	1.77	2.47
	B	0.86	4.74	1.41	2.3	2.44	1.04	2.17
	C	1.11	7.79	1.55	3.24	4.55	1.38	2.78
	D	1.19	6.57	1.44	2.97	3.61	1.43	2.28
	E	2.33	16.64	1.58	7.92	8.72	2.94	2.86
T6	A	5.42	43.98	1.84	17.91	26.07	6.96	4.38
	B	5.77	33.74	1.42	15.75	17.991	6.84	2.27
	C	2.02	17.24	1.91	4.9	12.34	2.59	5.07
	D	11.36	56.95	1.3	23.51	33.45	12.96	1.86
	E	2.72	25.12	1.61	11.95	13.16	3.4	3.19

**Table A2:** Parameters of roughness for input site obtained using a contact profilometer.

Sample Number	Measuring Area	Ra [µm]	Rz [µm]	Rsk	Rp [µm]	Rv [µm]	Rq [µm]	Rku
T1	A	0.65	8.85	2.09	5.24	3.62	0.88	6.02
	B	0.76	8.78	1.77	4.05	4.73	0.98	3.86
	C	0.81	11.64	2.02	7.28	4.36	1.09	5.52
	D	0.94	10.55	2.04	6.16	4.5	1.32	5.18
	E	0.85	9.62	1.7	4.89	4.73	1.08	3.53
T2	A	0.4	4.12	1.7	1.88	2.24	0.52	3.46
	B	0.61	6.91	1.78	3.43	3.49	0.79	3.91
	C	0.38	4.62	1.83	2.43	2.19	0.49	4.28
	D	0.63	5.13	1.55	2.68	2.45	0.79	2.77
	E	0.88	8.57	1.72	4.51	4.06	1.13	3.59
T3	A	0.39	9.75	2.83	4.22	5.53	0.57	13.08
	B	0.65	8.19	1.98	3.97	4.22	0.93	4.68
	C	1.21	21.76	2.31	10.2	11.55	1.74	7.34
	D	0.74	8.46	1.82	4.38	4.08	1.02	3.89
	E	0.75	8.06	1.87	3.87	4.19	1.07	4.06
T4	A	0.53	9.5	2.42	5.52	3.98	0.81	7.56
	B	0.83	9.08	1.99	4.62	4.46	1.22	4.59
	C	0.71	11.94	2.09	8.09	3.85	1.02	5.63
	D	0.9	10.23	2.13	4.81	5.42	1.41	5.13
	E	0.74	5.64	1.5	3.16	2.48	0.92	2.54
T5	A	0.26	5.95	3.09	3.12	2.83	0.41	13.41
	B	0.41	3.32	1.68	1.75	1.57	0.53	3.3
	C	0.32	4.8	2.09	2.66	2.14	0.45	5.62
	D	0.56	5.02	1.83	2.74	2.28	0.78	3.86
	E	0.3	4.46	1.7	2.55	1.91	0.38	3.74
T6	A	0.6	15.91	3.4	8.43	7.48	1.04	15.81
	B	0.79	17.8	2.36	8.78	9.02	1.14	8.51
	C	0.48	9.73	2.22	4.48	5.25	0.66	7.29
	D	1.3	16.32	1.85	8.91	7.41	1.79	4.14
	E	0.86	16.35	2.5	10.85	5.5	1.24	9.73
